# Maturation of Plastid *c*-type Cytochromes

**DOI:** 10.3389/fpls.2017.01313

**Published:** 2017-07-26

**Authors:** Stéphane T. Gabilly, Patrice P. Hamel

**Affiliations:** ^1^Department of Molecular Genetics and Department of Biological Chemistry and Pharmacology, The Ohio State University, Columbus OH, United States; ^2^Molecular and Cellular Developmental Biology Graduate Program, The Ohio State University, Columbus OH, United States

**Keywords:** thylakoid lumen, photosynthesis, cytochrome *c*, heme, thioether, cytochrome *b*_6_*f*

## Abstract

Cytochromes *c* are hemoproteins, with the prosthetic group covalently linked to the apoprotein, which function as electron carriers. A class of cytochromes *c* is defined by a C*XX*CH heme-binding motif where the cysteines form thioether bonds with the vinyl groups of heme. Plastids are known to contain up to three cytochromes *c*. The membrane-bound cytochrome *f* and soluble cytochrome *c*_6_ operate in photosynthesis while the activity of soluble cytochrome *c*_6A_ remains unknown. Conversion of apo- to holocytochrome *c* occurs in the thylakoid lumen and requires the independent transport of apocytochrome and heme across the thylakoid membrane followed by the stereospecific attachment of ferroheme via thioether linkages. Attachment of heme to apoforms of plastid cytochromes *c* is dependent upon the products of the *CCS* (for **c**ytochrome ***c*** **s**ynthesis) genes, first uncovered via genetic analysis of photosynthetic deficient mutants in the green alga *Chlamydomonas reinhardtii*. The CCS pathway also occurs in cyanobacteria and several bacteria. CcsA and CCS1, the signature components of the CCS pathway are polytopic membrane proteins proposed to operate in the delivery of heme from the stroma to the lumen, and also in the catalysis of the heme ligation reaction. CCDA, CCS4, and CCS5 are components of trans-thylakoid pathways that deliver reducing equivalents in order to maintain the heme-binding cysteines in a reduced form prior to thioether bond formation. While only four CCS components are needed in bacteria, at least eight components are required for plastid cytochrome *c* assembly, suggesting the biochemistry of thioether formation is more nuanced in the plastid system.

## Cytochromes of the *c*-Type

Cytochromes of the *c*-type, often generically referred to as cytochromes *c*, are membrane-bound or soluble metalloproteins occurring in energy-transducing membranes in archaea, bacteria, mitochondria, and plastids, where they function as electron carriers in respiration and photosynthesis (Thony-Meyer, 1997; Bonnard et al., 2010; Kletzin et al., 2015). Cytochromes *c*, on the positive side (or *p*-side)^1^ of energy-transducing membranes, carry one or several hemes (ferroprotoporphyrin IX) as a prosthetic group covalently attached via thioether bonds to a heme binding site in the apoprotein. The most common heme binding site consists of a C*XX*CH motif where the first and second cysteines are, respectively, linked to the vinyl-2 and vinyl-4 groups of heme and the intervening residue *X* can be any amino-acid except cysteine in naturally occurring cytochromes *c* (Allen et al., 2004; Bowman and Bren, 2008). The histidine residue serves as the proximal axial ligand of the iron atom. A distant histidine, methionine, or, less commonly, other residues in the apocytochrome provide distal axial ligation of the heme group (Bowman and Bren, 2008).

Variations of the heme binding site are rare and one example is the A/FXXCH motif of mitochondrial cytochromes *c* in Euglenozoa that bind the vinyl-4 group of heme via a single thioether bond (Priest and Hajduk, 1992; Fülöp et al., 2009). Other non-canonical heme binding sites occur in bacterial cytochromes *c* and contain three, four, or fifteen intervening residues between the cysteines instead of two (Herbaud et al., 2000; Aragão et al., 2003; Hartshorne et al., 2007) or a lysine instead of a histidine as the proximal heme ligand (Einsle et al., 1999). Another atypical cytochrome *c* is also the only known example of a cytochrome *c* on the negative side (*n*-side)^2^ of the membrane. This is cytochrome *b*_6_ of the *b*_6_*f* complex in plastids and cyanobacteria and cytochrome *b* of the *bc* complex in firmicutes, which contain a heme covalently attached via a single thioether bond (de Vitry, 2011). The heme binding cysteine faces the *n*-side of the membrane and is not found in a motif, unlike other *p*-side localized cytochromes *c*. Moreover, there are no amino-acid residues serving as proximal and distal ligands of heme, differentiating this cytochrome *c* from all other *c*-type cytochromes occurring on the *p*-side (de Vitry, 2011).

## Cytochrome *c* Maturation Systems

All *p*-side localized holocytochromes *c* are assembled on the *p*-side of the membrane. This requires the apoform and the heme moiety, both of which are transported independently across at least one biological membrane. Conversion of apocytochrome to its holoform requires free sulfhydryls at the C*XX*CH motif, provision of heme under the reduced form (ferroheme), and stereospecific attachment of the prosthetic group via catalysis of the thioether bond linkage (Mavridou et al., 2013; Travaglini-Allocatelli, 2013). Extensive genetic and biochemical analyses in bacteria, plants, and fungi revealed that the operation of three distinct assembly pathways called Systems I, II, and III is required for cytochrome *c* maturation, depending on the location (Kranz et al., 2009; Mavridou et al., 2013; Travaglini-Allocatelli, 2013; Verissimo and Daldal, 2014; Babbitt et al., 2015). The diversity of maturation systems is surprising, considering the biochemical requirements for heme attachment to apocytochrome *c* are believed to be universal and thioether bond formation appears, *a priori*, a simple chemical reaction (Bowman and Bren, 2008). Each System can be recognized by prototypical assembly factors but the number of such assembly factors and their features differ considerably among the different Systems (**Table 1**). An additional layer of complexity is the apparent “mosaic” distribution of Systems I, II, and III among organisms and the different energy transducing membranes (Mavridou et al., 2013; Travaglini-Allocatelli, 2013). Several evolutionary scenarios accounting for the origin and distribution of the different maturation systems have been proposed but the complexity of cytochrome *c* maturation as a biochemical process still remains mysterious (Bertini et al., 2007; Allen et al., 2008; Giegé et al., 2008; Kranz et al., 2009; Allen, 2011).

**Table 1:** Prototypical components of cytochrome *c* maturation pathways.

Function/Activity	System I	System II		System III
		Bacteria	Plastids	
Transmembrane heme transport	?	CcsA, CcsB^4^	CCS1, CcsA	?
Heme handling	CcmABCDE^1^	CcsA, CcsB	CCS1, CcsA	HCCS
Heme reduction	CcmF^2^	?	?	?
Apocytochrome c chaperoning	CcmH^3^	CcsB	CCS1	HCCS
Maintenance of reduced CXXCH sulfhydryls	DsbD CcmG	DsbD CcsX	CCDA CCS5 CCS4	?
Thioether bond formation	CcmFH	CcsA, CcsB	CCS1, CcsA	HCCS
Unknown			CCS2, CCS3, CCS6	

*The prototypical components of Systems I, II, and III are indicated according to their proposed function in the maturation process. Bacterial cytochromes *c* are assembled via System I or II. Plastid cytochrome *c* assembly relies on System II and mitochondrial cytochromes *c* are matured via System I or III. System III is restricted to mitochondria and is defined by a single component, holocytochrome *c* synthase (HCCS). The nomenclature for *Escherichia coli* (System I), *Bordetella pertussis* (System II bacteria), and *Chlamydomonas reinhardtii* (System II plastids) is used here. With the exception of System II, for which a heme transport across the membrane is supported by experimental evidence, there is no description of transmembrane heme delivery routes in Systems I and III. ‘?’ indicates that there is no component identified for this activity in the maturation process. *System I*: CcmABCDE^1^ is a periplasmic heme handling route defined by an ABC transporter (CcmAB), a member of the Heme Handling Protein (HHP) family (CcmC), and a small transmembrane protein (CcmD) that are required to load heme onto a heme chaperone (CcmE) (Kranz et al., 2009). CcmF^2^ and thiol-disulfide oxidoreductase CcmH^3^ are forming a complex postulated to carry the holocytochrome *c* synthase activity (Kranz et al., 2009). *System II:* CcsB^4^ is the bacterial ortholog of plastid CcsA*.

## Plastid *c*-Type Cytochromes

Three *c*-type cytochromes, have been identified within the thylakoid lumen of various plastids: the membrane-bound cytochrome *f* and the soluble cytochromes *c*_6_ and *c*_6A_. While cytochrome *f* and *c_6_* are known to function as electron carriers in photosynthesis, cytochrome *c*_6A_ function remains enigmatic despite having been discovered 15 years ago (Howe et al., 2006). All plastid cytochromes *c* contain a single heme attached to a C*XX*CH motif on the apoprotein. Cytochrome *f*, a catalytic subunit of the cytochrome *b*_6_*f* complex, is universal in all photosynthetic eukaryotes (and cyanobacteria) and is essential for photosynthesis (Martinez et al., 1994). Cytochrome *c*_6_ is found in cyanobacteria and the plastid of eukaryotic algae, where it is widely distributed among green, red and brown algal lineages (Sandmann et al., 1983; Kerfeld and Krogmann, 1998). Cytochrome *c*_6_ is involved in the transfer of electrons from cytochrome *f* of the cytochrome *b*_6_*f* complex to Photosystem I (Merchant and Dreyfuss, 1998). In green algae and cyanobacteria, cytochrome *c*_6_ acts as a substitute for plastocyanin in Cu-deficient conditions (Merchant and Bogorad, 1987a,b). Cytochrome *c*_6A_ occurs in the thylakoid lumen of land plants and green algae but appears absent in red algae and diatoms (Wastl et al., 2004).

Cytochrome *c*_6A_ was discovered in *Arabidopsis* as a protein interacting with the lumen-localized immunophilin FKBP13 in a yeast two-hybrid screen (Gupta et al., 2002a,b; Buchanan and Luan, 2005). It was initially postulated that cytochrome *c*_6A_ acts as a substitute for plastocyanin (Gupta et al., 2002a), as in green algae and cyanobacteria where cytochrome *c*_6_ can replace plastocyanin (Merchant and Bogorad, 1987a,b). However, loss of cytochrome *c*_6A_ in *Arabidopsis* has no visible phenotype even under Cu deficient conditions (Gupta et al., 2002a). Moreover, an *Arabidopsis* plastocyanin-deficient mutant is unable to grow photoautotrophically even when cytochrome *c*_6A_ is overexpressed (Weigel et al., 2003). *In vitro*, cytochrome *c*_6A_ is unable to provide electrons to Photosystem I (Molina-Heredia et al., 2003). This observation accounts for the fact that cytochrome *c*_6A_ cannot act as a functional substitute for plastocyanin *in vivo*. Hence, cytochrome *c*_6A_ does not appear to function in the known electron transfer reactions of photosynthesis, which is consistent with its extremely low abundance in the thylakoid. The presence of a disulfide bond in holocytochrome *c*_6A_ led to the proposal that the molecule acts as an oxidant of luminal proteins dithiols with heme providing electrons for re-oxidation of the cysteine pair (Marcaida et al., 2006). Additional experimental exploration is required to test this hypothesis.

## System II, A Multicomponent Assembly Pathway Required for Maturation of Plastid Cytochromes *c*

Plastid cytochromes *c* are matured via System II, also referred to as the CCS pathway, a multicomponent assembly machinery (Hamel et al., 2009; Bonnard et al., 2010; Simon and Hederstedt, 2011). System II first emerged through genetic screens for photosynthesis-impaired *ccs* mutants (*ccs* for *c*ytochrome *cs*ynthesis) in the green alga *Chlamydomonas reinhardtii* (Hamel et al., 2009; Simon and Hederstedt, 2011). The *Chlamydomonas ccs* mutants were isolated on the basis of a dual deficiency in the holoforms of both cytochrome *f* and cytochrome *c*_6_. All *ccs* mutants are photosynthetic deficient because loss of cytochrome *f* assembly results in a *b*_6_*f*-minus phenotype (Howe and Merchant, 1992; Inoue et al., 1997; Xie et al., 1998; Dreyfuss and Merchant, 1999; Page et al., 2004). Pulse-chase experiments revealed that both plastid apocytochromes *c* are synthesized, imported in the thylakoid lumen, and processed by lumen-resident signal peptidase, but they fail to be converted to their respective holoforms (Howe and Merchant, 1993, 1994; Xie et al., 1998). Based on these experiments, it was concluded the *ccs* mutants exhibit a defect in the heme attachment to apoforms of cytochrome *c* in the thylakoid lumen (Howe and Merchant, 1993, 1994; Xie et al., 1998). The *ccs* mutants are also expected to display a defect in cytochrome *c*_6A_. However, this could not be tested because holocytochrome *c*_6A_ could not be detected in a wild-type strain. The defect in the *ccs* mutants is specific to plastid *c*-type cytochromes since plastocyanin, another lumen-resident metalloprotein, is normally assembled (Howe and Merchant, 1993, 1994; Xie et al., 1998). The *ccs* mutants are not affected for the covalent attachment of heme to the single *n*-side facing cysteine in cytochrome *b*_6_, a structural subunit of the *b*_6_*f* complex (Kuras et al., 2007). Catalysis of this thioether bond in cytochrome *b*_6_, occurs on the stromal side of the thylakoid membrane and is dependent upon the *CCB* gene products (de Vitry, 2011).

## CcsA and CCS1, A Heme Delivery Complex with Holocytochrome *c* Synthase Activity

**FIGURE 1:**
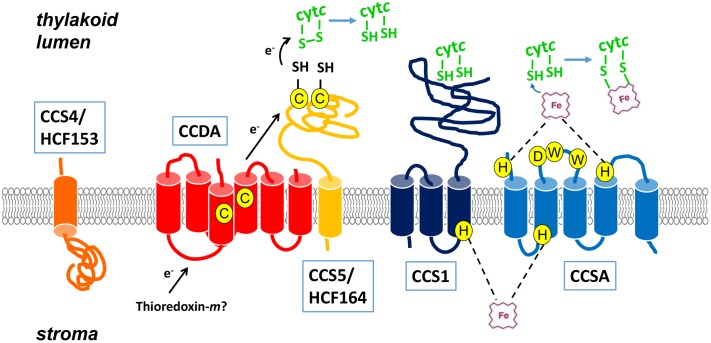
A Model for plastid cytochrome *c* maturation. Plastid cytochrome *c* maturation involves two different pathways: (1) a *trans*-thylakoid disulfide reducing route with CCDA (red), CCS5/HCF164 (light orange), and CCS4/HCF153 (orange). The CCDA topology was deduced using PhoA/LacZ topological reporters (Page et al., 2004). CCS5/HCF164 is a membrane anchored, lumen-facing, thioredoxin-like protein (Lennartz et al., 2001). Cysteines (C) are circled and highlighted in yellow. Electron (e^-^) transfer is indicated by black arrows. Stromal thioredoxin-*m* is the possible reductant for the CCDA-CCS5/HCF164 pathway, which conveys electrons to reduce disulfide-bonded C*XX*CH motif in apocytochrome *c*. CCS4/HCF153 is bound to the thylakoid membrane (Lennartz et al., 2006) and the soluble part of the protein is predicted to be facing the stroma based on the positive-inside rule (Gabilly et al., 2011). The role of CCS4/HCF153 in cytochrome *c* biogenesis is unclear, but it may be involved in the transport of reductant across the membrane. (2) A heme delivery/cytochrome *c* synthase pathway composed of CCS1 (dark blue) and CcsA (light blue). CcsA and CCS1 topologies were deduced using PhoA/LacZ topological reporters (Hamel et al., 2003; Dreyfuss et al., 2003). The CCS1/CcsA complex binds and channels the reduced heme from the stroma to the thylakoid lumen and catalyzes thioether bond formation. Two histidines, one from CCS1 and one from CcsA, on the stromal side of the thylakoid membrane could function in relaying the heme across the membrane. Two histidines in CcsA on the luminal side could act by coordinating heme that interacts with the WWD domain. Strictly conserved histidines (H), tryptophans (W), and asparatic acids (D) are circled and highlighted in yellow. The thylakoid membrane is drawn in gray.

The first CCS component to be identified is plastid-encoded CcsA (Xie and Merchant, 1996; Hamel et al., 2003), a thylakoid membrane protein belonging to the HHP (Heme Handling Protein) superfamily (Lee et al., 2007), which is defined by the highly conserved tryptophan-rich WWD motif and conserved histidine residues (**Figure 1**). This feature is also shared by CcmC and CcmF, two HHPs in System I shown to relay heme on the bacterial periplasmic space (Richard-Fogal et al., 2009; Richard-Fogal and Kranz, 2010) (**Table 1**). The other prototypical component is CCS1, a thylakoid membrane protein with little sequence conservation and lacking domains or structural features speaking to a specific chemical function, with the exception of an invariant histidine (Inoue et al., 1997). Because all photosynthetic plastid genomes (with a few exceptions) encode a CcsA-like protein, the CCS pathway is believed to operate in the plastids of all photosynthetic eukaryotes. System II also occurs in cyanobacteria, a majority of the Gram-positive bacteria, proteobacteria of the β-, δ-, and ε groups, and aquificales (Hamel et al., 2009; Bonnard et al., 2010; Simon and Hederstedt, 2011).

Detailed studies, including topological studies of algal CcsA and cyanobacterial Ccs1, site-directed mutagenesis of conserved residues, and molecular analysis of existing *ccs1* alleles, established that CcsA and CCS1 are polytopic membrane proteins with functional domains exposed to the lumen and four strictly conserved essential histidine residues on both the lumen and stromal sides (Dreyfuss et al., 2003; Hamel et al., 2003) (**Figure 1**). Functional domains include the WWD signature motif for CcsA and a large hydrophilic C-terminal extension for CCS1. The C-terminal domain was postulated to chaperone apocytochrome *c* from studies of cyanobacterial Ccs1 (Tichy and Vermaas, 1999). In *Chlamydomonas*, a 200 kDa CCS1-containing complex in wild-type no longer accumulated in a *ccsA* mutant, suggesting that CcsA and CCS1 occur in a complex. The reduced abundance of CCS1 in some *ccs* mutants suggests the 200 kDa complex may contain other CCS components besides CcsA and CCS1 (Hamel et al., 2003). This led to the proposal that these two proteins act together to relay heme via histidinyl coordination from its site of synthesis, the stroma, to the lumen. In the lumen, heme is relayed to the WWD domain and coordinated by two histidine residues in CcsA (Hamel et al., 2003; **Figure 1**).

Experimental proof that CcsA and Ccs1 catalyze the heme attachment reaction was provided with the finding that Ccs1-CcsA fusion proteins, naturally occurring in several ε-proteobacteria, could assemble reporter cytochrome(s) *c* in an *Escherichia coli* strain lacking its endogenous cytochrome *c* assembly machinery (Feissner et al., 2006; Frawley and Kranz, 2009; Goddard et al., 2010; Kern et al., 2010; Richard-Fogal et al., 2012). Biochemical evidence supporting a possible role of CcsA and Ccs1 in heme transport from the cytoplasm to the periplasm came from studies of the Ccs1-CcsA fusion from *Helicobacter hepaticus*. Spectroscopic analysis of the recombinant fusion protein identified the presence of heme. Mutagenesis of the two periplasm-facing histidines highlighted the importance of these residues for the binding of heme and its maintenance in a reduced state (Frawley and Kranz, 2009). This led to the hypothesis that CcsA carries a heme binding site on the periplasmic space, presumably required for the cytochrome *c* synthase activity. To test the function of the two transmembrane cytoplasm-facing histidines in Ccs1 and CcsA, these residues were mutated in the recombinant protein. Because heme is synthesized in the cytoplasm and was no longer detected in the mutated form of the protein, it was concluded that these histidines provide an entry site for heme through the lipid bilayer on the cytoplasmic side of the membrane.

This implied that Ccs1-CcsA functions in channeling heme from the cytoplasm to the periplasm, but a direct heme transport activity remains to be demonstrated. By analogy, it is plausible that plastid CcsA and CCS1 also function in a heme relay pathway from stroma to lumen and carry the cytochrome *c* synthase activity but this has not been tested. While candidate components for the chemical reduction of heme were identified in System I, it is unknown how this process is achieved in System II (**Table 1**).

## Operation of *Trans*-Thylakoid Disulfide Reducing Pathways

The operation of a thylakoid transmembrane thiol-disulfide relay in plastid cytochrome *c* maturation emerged with the description of two thiol-disulfide oxidoreductases at the thylakoid membrane, namely CCDA, a member of the DsbD family, and HCF164, a membrane-anchored, lumen-facing protein that displays similarity to thioredoxin-like CcmG and CcsX (**Table 1**) (Lennartz et al., 2001; Page et al., 2004; Motohashi and Hisabori, 2006; Motohashi and Hisabori, 2010). In bacteria using Systems I and II, cytochrome *c* maturation requires the provision of reductants via sequential thiol-disulfide exchanges involving a cytoplasmic thioredoxin, a thiol-disulfide reductase of the DsbD family, and a periplasmic thioredoxin-like protein (CcmG in System I or CcsX in System II) (**Table 1**) (Mavridou et al., 2013; Travaglini-Allocatelli, 2013). The working model is that the apocytochrome *c* C*XX*CH motif is first disulfide bonded by the disulfide bond forming enzymes residing in the periplasm and subsequently reduced by a thioredoxin-like protein (CcmG or CcsX) dedicated to the heme attachment reaction (Mavridou et al., 2013; Travaglini-Allocatelli, 2013). Reverse-genetic analysis in *Arabidopsis* indicates a function for CCDA and HCF164 in holocytochrome *f* accumulation, but a possible defect in the heme attachment reaction was not investigated (Lennartz et al., 2001; Page et al., 2004).

The biochemical requirement for thiol-disulfide chemistry in plastid cytochrome *c* biogenesis was demonstrated with the identification of CCS5, the *Chlamydomonas* ortholog of thioredoxin-like HCF164 (Gabilly et al., 2010). CCS5 physically interacts with plastid apocytochromes *c* and a recombinant form of the CCS5 molecule is active as a reductase when apocytochrome *c* with a disulfide-bonded C*XX*CH motif is provided as a substrate in an *in vitro* reaction (Gabilly et al., 2010). Application of exogenous thiols to the *ccs5*-null mutant rescues the photosynthetic deficiency and holocytochrome *f* assembly, an indication that CCS5 acts as a disulfide reductase *in vivo* (Gabilly et al., 2010). By analogy to the bacterial pathway, CCS5/HCF164 is likely to be maintained reduced by the activity of CCDA but this remains to be experimentally tested (**Figure 1**). The source of reducing equivalents on the stromal side was attributed to thioredoxin-*m* (Trx-*m*) (**Figure 1**) based on the observation that the redox active cysteines in CCDA and HCF164 undergo reduction in isolated thylakoid membranes when Trx-*m* is added exogenously (Motohashi and Hisabori, 2010). Complete loss of function of CCDA or HCF164 in *Arabidopsis* and CCS5 in *Chlamydomonas* does not abolish plastid cytochrome *c* maturation, an indication that another mechanism for delivery of reductant must exist (Lennartz et al., 2001; Page et al., 2004; Gabilly et al., 2010).

Evidence of an additional pathway for the supply of reducing power was provided with the finding that the *ccs4* mutant is restored for cytochrome *c* assembly by application of exogenous thiols (Gabilly et al., 2011). CCS4 is a small protein with an N-terminal membrane anchor and a C-terminal domain predicted to be exposed to the stromal side of the thylakoid membrane but does not display any motif or residue (such as cysteines) suggesting a role in thiol-based redox chemistry (Gabilly et al., 2011). CCS4 exhibits similarity to *Arabidopsis* HCF153, a thylakoid membrane anchored protein with a stromal facing C-terminal domain required for cytochrome *b*_6_*f* accumulation (Lennartz et al., 2006). In addition to the thiol-dependent photosynthetic rescue of the *ccs4* mutant, the placement of CCS4 in a disulfide-reducing pathway for cytochrome *c* assembly is further substantiated by the fact that ectopic expression of CCDA, a thiol/disulfide oxidoreductase of the DsbD family, at the thylakoid membrane suppresses the *ccs4* mutant (Gabilly et al., 2011). As none of the *CCS* loci correspond to *CCDA* (Page et al., 2004), the CCDA-dependent suppression of the *ccs4* mutant provides indirect evidence for the function of CCDA in plastid cytochrome *c* maturation. The suppression can be explained by a compensatory effect due to enhanced expression of the thiol-disulfide oxidoreductase CCDA. The activity of CCS4 in the heme attachment reaction so far remains unclear but one attractive scenario is that it controls the delivery of reducing power through the membrane via transport of a reductant. There is precedence for this in bacterial periplasm where reducing power, in the form of cysteine or glutathione, is transferred from the cytoplasm to the periplasm via specific transporters (Pittman et al., 2005; Ohtsu et al., 2010).

## Other CCS Components Unique to Plastid Cytochrome *C* Maturation

In bacteria using the CCS pathway, CcsA, Ccs1, a thiol-disulfide reductase of the DsbD family, and a thioredoxin-like protein are the only components required to complete holocytochrome *c* assembly (Beckett et al., 2000; Le Brun et al., 2000). From the genetic analysis of the *Chlamydomonas ccs* mutants, it appears that cytochrome *c* maturation in plastids is a more complicated process than in bacteria. This seems counter-intuitive considering that bacteria can assemble numerous mono and multiheme cytochromes *c* via the CCS pathway, while plastids only need to mature up to three monoheme cytochromes. In addition to CcsA, CCS1, CCDA, and HCF164/CCS5, plastid cytochrome *c* maturation also requires CCS4 and the products of the *CCS2*, *CCS3*, and *CCS6* genes (Xie et al., 1998; Page et al., 2004), which remain uncharacterized. The fact that single alleles map to the *Chlamydomonas CCS3, CCS4, CCS5*, and *CCS6* loci suggests that mutant screens for plastid cytochrome *c* deficient mutants are not saturated and additional CCS loci could still be uncovered (Howe and Merchant, 1992; Xie et al., 1998; Dreyfuss and Merchant, 1999; Page et al., 2004).

## Author Contributions

SG and PH wrote the manuscript jointly. SG designed the figure.

## Conflict of Interest Statement

The authors declare that the research was conducted in the absence of any commercial or financial relationships that could be construed as a potential conflict of interest.

## Footnotes

1 The *p*-side corresponds to the bacterial or archaeal periplasm, the thylakoid lumen and the mitochondria intermembrane space.

2 The *n*-side corresponds to the bacterial or archaeal cytoplasm, the plastid stroma and the mitochondrial matrix.
